# The Association Between Folate and Alzheimer's Disease: A Systematic Review and Meta-Analysis

**DOI:** 10.3389/fnins.2021.661198

**Published:** 2021-04-14

**Authors:** Xiaohong Zhang, Guangyi Bao, Debiao Liu, Yu Yang, Xuezhi Li, Gaomei Cai, Yan Liu, Yili Wu

**Affiliations:** ^1^College of Marine Life Sciences, Ocean University of China, Qingdao, China; ^2^Shandong Key Laboratory of Behavioral Medicine, School of Mental Health, Jining Medical University, Jining, China; ^3^Shandong Collaborative Innovation Center for Diagnosis, Treatment & Behavioral Interventions of Mental Disorders, Institute of Mental Health, Jining Medical University, Jining, China; ^4^Center of Evidence-Based Medicine, Jining Medical University, Jining, China; ^5^Department of Neurology, Affiliated Hospital of Jining Medical University, Jining Medical University, Jining, China

**Keywords:** Alzheimer's disease, folate level, folate deficiency, sufficient folate intake, meta-analaysis

## Abstract

Alzheimer's disease (AD) is the most common type of neurodegenerative disease leading to dementia in the elderly. Increasing evidence indicates that folate plays an important role in the pathogenesis of AD. To investigate the role of folate deficiency/possible deficiency in the risk of AD and the benefical effect of sufficient folate intake on the prevention of AD, a systematic review and meta-analysis were performed. The Web of Science, PubMed, CENTRAL, EBSCO, CNKI, CQVIP, and Wanfang databases were searched. The analysis of cross-sectional studies showed that the standardized mean difference (SMD) was −0.60 (95% confidence interval (CI): −0.65, −0.55), indicating that plasma/serum folate level is lower in AD patients than that in controls. Moreover, the combined odds ratio (OR) of case-control studies was 0.96 (95% CI: 0.93, 0.99), while the combined ORs were 0.86 (95% CI: 0.46, 1.26) and 1.94 (95% CI: 1.02, 2.86) in populations with normal levels of folate (≥13.5 nmol/L) and folate deficiency/possible deficiency (<13.5 nmol/L), respectively. In addition, the risk ratio (RR) of the cohort studies was 1.88 (95% CI: 1.20, 2.57) in populations with folate deficiency/possible deficiency. Furthermore, when the intake of folate was equal to or higher than the recommended daily allowance, the combined RR and hazard ratio (HR) were 0.44 (95% CI: 0.18, 0.71) and 0.76 (95% CI: 0.52, 0.99), respectively. These results indicate that folate deficiency/possible deficiency increases the risk for AD, while sufficient intake of folate is a protective factor against AD.

## Introduction

Alzheimer's disease (AD) is the most common type of neurodegenerative disease leading to dementia in the elderly. A progressive memory loss and deterioration of other cognitive functions are the main clinical manifestations, while extraneuronal neuritic plaques, intraneuronal neurofibrillary tangles, and neuronal loss are the neuropathological hallmarks of AD (Hebert et al., 2013; Bakota and Brandt, 2016; Mantzavinos and Alexiou, 2017; Li et al., 2018). According to the age of onset, AD is classified into early-onset AD (EOAD) and late-onset AD (LOAD). Compared with EOAD (onset before age 65), LOAD (onset after age 65) accounts for 95% or more of AD cases (Alzheimer's Association, 2012). With the rapid increase of the aging population worldwide, over 50 million people were living with dementia globally in 2019 and the number is said to increase to 152 million by 2050 (Alzheimer's Disease International, 2019). The total cost for dementia was about 1 trillion US dollars in 2019 and it will be doubled by 2030 (Alzheimer's Disease International, 2019). AD accounts for 60–80% of dementia. Preventing or delaying the onset of AD is a priority as there is no effective treatment for AD.

Increasing evidence has indicated that dietary patterns and nutrition are implicated in the pathogenesis of AD (Otaegui-Arrazola et al., 2014). Thus, healthy diet and the balance of nutrients including vitamins are key factors in AD prevention. For example, marginal vitamin A deficiency promotes Aβ generation, the major component of neuritic plaques, and subsequent cognitive deficits (Zeng et al., 2017). Increasing evidence suggests that folate, an essential vitamin, plays an important role in AD development (Jheng et al., 2016; Tian et al., 2016; Robinson et al., 2018; Guo et al., 2019). The normal range of plasma/serum folate ranges from 13.5 nmol/L to 45.3 nmol/L. Folate deficiency and possible deficiency are defined when the level of plasma/serum folate is <6.8 nmol/L and 13.5 nmol/L, respectively (WHO, 2015). Previous studies showed that low folate level is not only associated with specific domains of cognitive functioning, e.g., episodic recall and recognition (Wahlin et al., 1996; Hassing et al., 1999; Nurk et al., 2005; De Lau et al., 2007), but also associated with all types of dementia, including vascular dementia and AD (Clarke et al., 1998; Ebly et al., 1998; Morris, 2003; Zhuo et al., 2011; Douaud et al., 2013; Cascalheira et al., 2015). In addition, folate/folic acid supplementation is beneficial to the improvement of cognitive functions in aged subjects and cases of mild cognitive impairment (Fioravanti et al., 1997; Morris, 2003; Durga et al., 2007; De Jager et al., 2012; Ma et al., 2019). Moreover, deprivation of folate increases tau phosphorylation, the major component of neurofibrillary tangles (Chan and Shea, 2006). However, there has not been a meta-analysis study to investigate the association between folate deficiency/possible deficiency and the risk of AD, as well as the benefical effect of sufficient folate intake on the prevention of AD.

This study aims to investigate the role of folate deficiency/possible deficiency in the risk of AD and the benefical effect of sufficient folate intake on the prevention of AD in addition to updating the association between plasma/serum folate levels and AD. We designed and performed this systematic review and meta-analysis to evaluate the difference of folate levels between AD patients and healthy controls, the association of folate deficiency/possible deficiency with AD risk, and the effect of sufficient folate intake on the prevention of AD.

## Materials and Methods

### Protocol and Registration

This systematic review and meta-analysis were conducted in accordance with the Meta-analysis Of Observational Studies in Epidemiology (MOOSE) (Stroup et al., 2000) statement published in 2009. The study protocol was developed before this review and was registered at PROSPERO with the registration number CRD42020173072.

### Search Strategy and Selection Criteria

To find publications of the association between folate and AD, two authors independently performed a systematic literature search in four English databases (Web of Science, PubMed, CENTRAL, EBSCO), and three Chinese databases (CNKI, CQVIP, and Wanfangdata). Boolean search techniques were carried out in full text, i.e., (Folic acid OR Folate OR vitamin B9 OR Vitamin Bc OR pteroylglutamic acid OR R factor OR MTHFR OR methyltetrahydrofolate reductase) AND (dementia OR Alzheimer). In addition, the references of identified publications were also screened by two independent authors. Only studies on AD, not other types of dementia [e.g., vascular dementia (VAD)], were included for the systematic review and meta-analysis in this study although both “dementia” and “Alzheimer” were included in the search terms list in order to avoid missing any study related to AD. The searching process was completed on December 31, 2019.

### Inclusion and Exclusion Criteria

The inclusion criteria were: (1) cross-sectional studies, case-control studies, or longitudinal studies with primary data collection; (2) studies conducted in the general population; (3) outcomes of individuals with AD were determined by clinicians' diagnosis according to the Diagnostic and Statistical Manual of Mental Disorders (DSM) criteria, the Consortium to Establish a Registry for Alzheimer's Disease (CERAD) criteria, the National Institute of Neurological and Communicative Disorders and Stroke and Alzheimer's Disease and Related Disorders Association (NINCDS-ADRDA) criteria or pathological criteria; (4) studies that provided sufficient statistical data to calculate the combined effect sizes; and (5) studies in any language and publication period.

The following types of studies were excluded: (1) studies with non-primary data such as opinion articles, editorials, letters to the editor, and comments; (2) animal studies; (3) qualitative studies; 4) dissertations; and (5) studies that could not provide sufficient statistical data.

### Quality Evaluation and Data Extraction

The Newcastle-Ottawa Scale (NOS, scores range from 0 to 9) was used to assess the quality and risk of bias (Stang, 2010). Studies with a NOS score of 6 or more were included for the meta-analysis. All discrepancies were resolved by discussion with a third author.

Two authors independently extracted the following data from all articles: first author's name, publication year, country of survey, number of cases and controls, mean age of participants, the mean and standard deviation of folate or odds ratio (OR) or risk ratio (RR) or hazard ratio (HR) and their 95% confidence intervals (CI).

The standardized mean difference (SMD) is used as a summary statistic in meta-analysis when the studies all assess the same outcome, but measure it in a variety of ways. The SMD expresses the size of the intervention effect in each study relative to the between-participant variability in outcome measurements observed in that study (Cochrane Training, 2020). In the current study, SMD was used to calculate differences of mean folate levels between people who did and did not suffer from AD. SMD and corresponding 95% CIs of plasma/serum folate were calculated based on the sample size, mean, and SD. Median and range were also used to estimate mean and SD (Hozo et al., 2005). 95% CI was transformed from SD through the formula as follows: 95% CI = mean ± 1.96 SD. The 25th and 75th percentiles were transformed to SD through the following formula: SD = Norm IQR = (P75–P25) ×0.7413 (IQR: inter-quartile range; P75: 75th percentile; P25: 25th percentile). SMD <0 represents a folate level that is lower in the AD group compared with that in the control group.

Measures of relative effect express the expected outcome in one group relative to that in the other. RR is the ratio of the risk of an event in the two groups, whereas OR is the ratio of the odds of an event. For both measures a value of 1 indicates that the estimated effects are the same for both interventions, while a value <1 might indicate a beneficial effect of an experimental intervention. The most appropriate way of summarizing time-to-event data is to use methods of survival analysis and express the intervention effect as a HR. Hazard is similar in notion to risk, but is subtly different in that it measures instantaneous risk and may change continuously (Cochrane Training, 2020). In the current study, OR, RR, and HR were used to indicate the beneficial effect (<1) or detrimental effect (>1) of different folate levels and amount of folate intake.

### Data Synthesis and Statistical Analysis

All statistical analyses were performed using Stata 15.0. The pooled effect size (SMD/OR/RR/HR) and its 95% CI were reported. A two-sided *p* <0.05 was considered statistically significant.

Different models were used based on heterogeneity tests. The heterogeneity of the included studies was evaluated using Higgins *I*^2^ test. The random-effect model was used if *I*^2^ >50%, which was marked in the forest plots. The fixed-effect model was used if *I*^2^ ≤ 50%, which was not marked in the forest plots.

Funnel plots and an Egger's test were used to investigate the potential publication bias. The Egger's test was only conducted when six or more studies were included.

### Sensitivity Analysis

When the heterogeneity was high (*I*^2^ >50%), sensitivity analysis was conducted to evaluate the stability of the outcome. Sensitivity analysis was performed by excluding an individual study at one time. A two-sided *p* <0.05 was considered statistically significant. The random-effect model was used. A subgroup meta-analysis was further conducted for the combined index of AD based on the level of plasma/serum folate and the daily intake of folate.

## Results

### Characteristics of Included Studies

**Figure 1:**
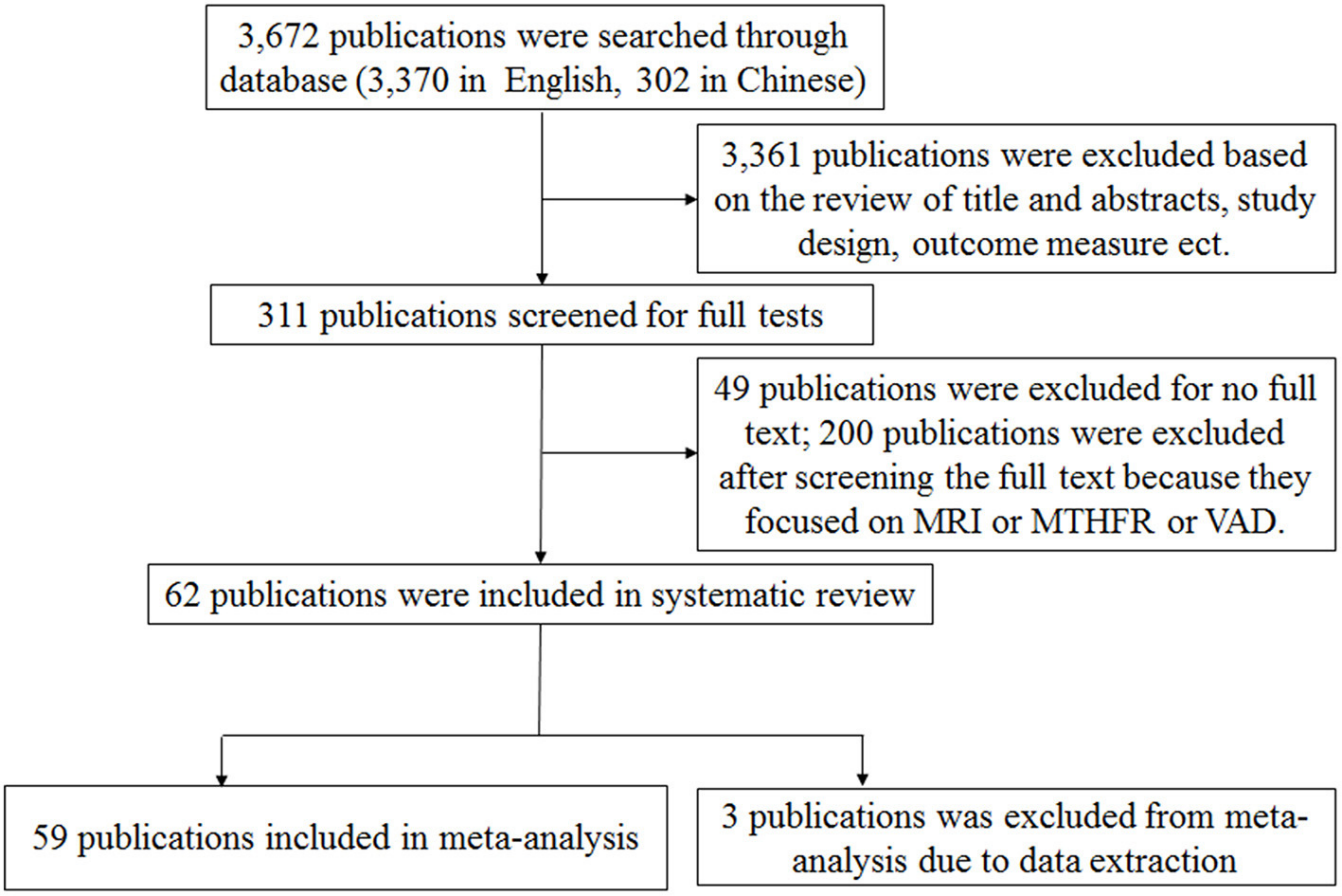
Flow diagram of the process used to breakdown the retrieved publications into publications suitable for meta-analysis.

A total of 3,672 publications (including 3,370 in English and 302 in Chinese) related to AD in the general population were initially identified from the databases. Sixty-two publications were included in this systematic review and 59 of them were included for further meta-analysis (Figure 1). Fifty-six and three articles were published in English and Chinese, respectively. Forty publications only included cross-sectional studies and eight publications only included cohort studies, respectively. Ten publications included both cross-sectional and case-control studies, while one publication included both cross-sectional and cohort studies. The studies were conducted in European countries (33 publications), Asian countries (14 publications), American countries (10 publications), and Oceania countries (2 publications), respectively.

Each subgroup of the analysis was considered as an independent study according to the methods previously used (Moazzen et al., 2018). In total, 52 cross-section studies, 14 case-control studies, and 16 cohort studies were included for meta-analysis. The sample size ranged from 24 to 965.

### The Level of Plasma/Serum Folate Is Lower in AD Patients: Results From Cross-Sectional Studies

**Figure 2:**
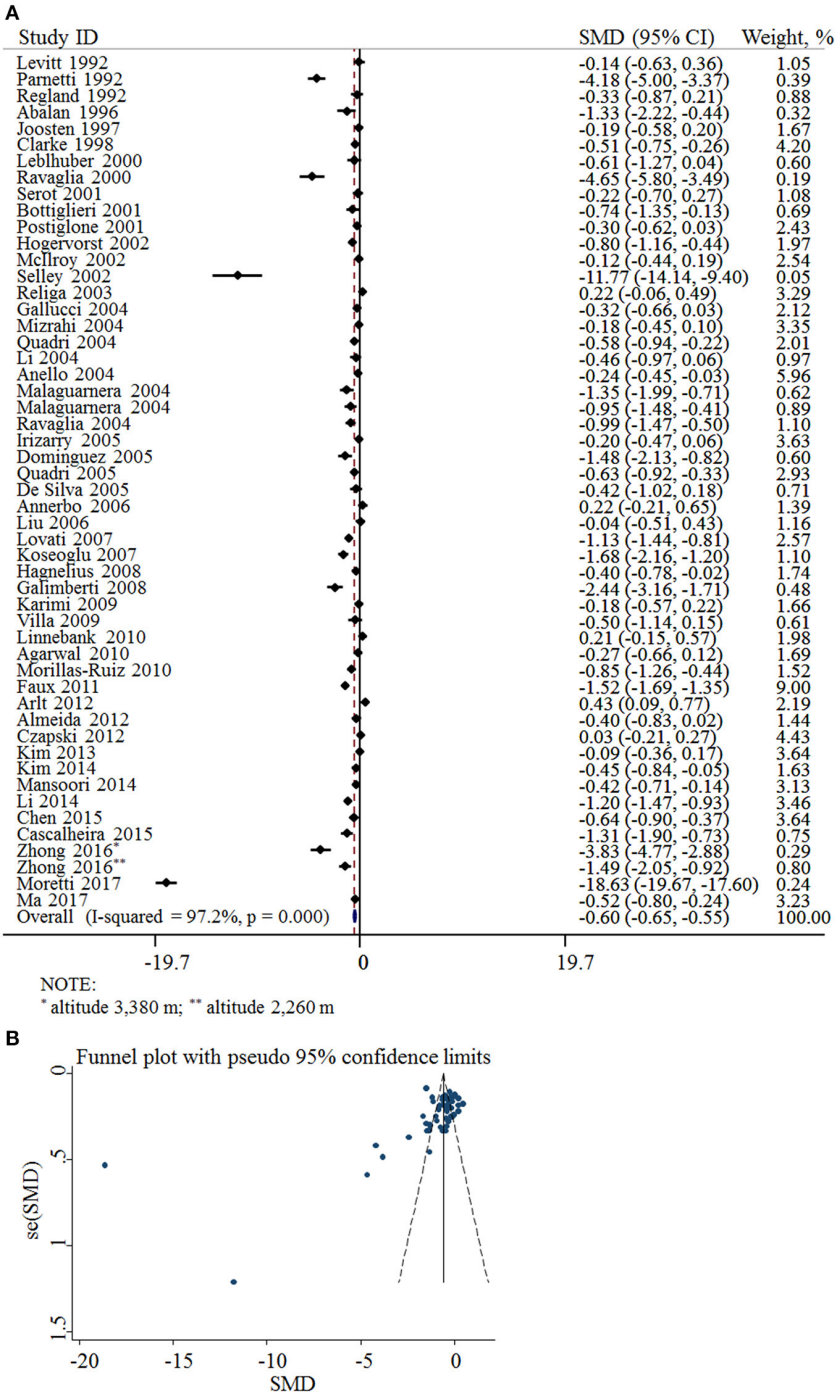
SMD analysis of the plasma/serum folate levels between AD and controls. **(A)** Pooled estimate of SMD and 95% CI of folate levels among AD patients and controls; **(B)** Funnel plot for publication bias of SMD.

Fifty-two cross-sectional studies covering a total of 3,496 AD patients and 4,318 controls were included in the meta-analysis. The summary of these studies is shown in Table 1. The level of plasma/serum folate was lower in AD patients than that in the controls, with an SMD of −0.60 (95% CI: −0.65, −0.55) (Figure 2A).

**Table 1:** Summary of studies of folate levels (nM/L) among AD patients and healthy controls in 52 cross-sectional studies.

**References**	**Mean age (years)**		**Sample size**		**Folate levels (nM/L)** **(Mean** **±** **SD)**		**Country**
	**AD**	**Control**	**AD**	**Control**	**AD**	**Control**	
Levitt and Karlinsky (1992)	68.4 ± 11.1	71.0 ± 9.9	40	26	15.5 ± 8.3	16.7 ± 9.6	Canada
Parnetti et al. (1992)	62.7 ± 1.2	72.1 ± 1.4	52	26	9.5 ±1.1	14.1 ± 1.1	Italy
Regland et al. (1992)	64 ± 5	65 ± 7	23	32	15.0 ± 10.0	20 ± 18.0	Sweden
Abalan et al. (1996)	80.2 ± 5.7	78.9 ± 7.2	12	12	10.19 ± 3.40	15.86 ± 4.98	French
Joosten et al. (1997)	82.8 ± 4.9	79 ± 5.9	52	49	7.9 ± 4.2	8.6 ± 3.2	Germany
Clarke et al. (1998)	73.2 ± 8.6	72.8 ± 8.8	164	108	17.6 ± 10.7	22.9 ± 10	UK
Leblhuber et al. (2000)	74.8 ± 8.8	70.2 ± 8.8	19	19	10 ± 3.4	14.3 ± 9.3	Austria
Ravaglia et al. (2001)	101 ± 2	103 ± 2	34	13	8.0 ± 0.5	11.5 ± 1.2	Italy
Serot et al. (2001)	75.9 ± 6.6	72.7 ± 7.02	30	36	12.11 ± 4.87	13.16 ± 4.82	France
Bottiglieri et al. (2001)	71 ± 8.5	40.6 ± 14.6	48	14	8.0 ± 3.4	12.1 ± 10	Italy
Postiglione et al. (2001)	68 ± 8	68 ± 7	74	74	5.7 ± 2.1	6.5 ± 3.2	Italy
Hogervorst and Smith (2002)	77 ± 8	76 ± 8	66	62	15.9 ± 11.3	24.9 ± 11.3	UK
Mcilroy et al. (2002)	77.2 ± 8.1	74.3 ± 7.6	83	71	10.65 ± 1.96	10.87 ±1.50	UK
Selley et al. (2002)	77.4	78.4	27	25	14.74 ± 0.82	25.09 ± 0.94	Australia
Religa et al. (2003)	74.2 ± 6.3	71.2 ± 6	99	100	19.3 ± 7.7	17.1 ± 12.2	Poland
Gallucci et al. (2004)	76.9 ± 6.8	76.9 ± 9.7	137	42	11.6 ± 6.1	14.0 ± 11.1	Italy
Mizrahi et al. (2004)	—	—	75	155	4.3 ± 3.2	4.8 ± 2.6	Israel
Quadri et al. (2004)	79.1 ± 7.7	75.6 ± 8.5	74	55	13.6 ± 5.6	16.9 ± 5.8	Italy
Peng et al. (2004)	69.4 ± 5	—	30	30	29.2 ± 12.7	37.2 ± 21.2	China
Anello et al. (2004)	71.0 ± 6.6	69.5 ± 12.7	180	181	14.3 ± 5.7	15.7 ± 5.9	Italy
Malaguarnera et al. (2004b)	72.6 ± 7.38	73.7 ± 4.20	22	24	10.0 ± 2.72	13.9 ± 3.03	Italy
Malaguarnera et al. (2004a)	71.3 ± 8.0	73.6 ± 4.1	30	30	10.6 ± 3.16	13.6 ± 3.18	Italy
Ravaglia et al. (2004)	86.7 ± 5.4	86.7 ± 5.9	51	29	11.1 ± 4.3	16.57 ± 7.26	Italy
Irizarry et al. (2005)	75.9 ± 8.7	70.3 ± 9.8	145	88	29.9 ± 21.3	35.2 ± 32.9	USA
Dominguez et al. (2005)	73.4 ± 5.4	73.9 ± 8.9	29	19	17.87 ± 7.18	29.57 ± 8.97	Argentina
Quadri et al. (2005)	78.9 ± 7.5	75.0 ± 8.5	111	79	13.1 ± 5.9	16.8 ± 5.5	Switzerland
De Silva et al. (2005)	72 ± 6.8	70.5 ± 3.9	23	21	15.9 ± 8.4	19.7 ± 9.7	Sri Lanka
Annerbo et al. (2006)	67.7 ± 7.2	63.6 ± 9.6	32	61	19.0 ± 14.0	16.4 ± 10.8	Sweden
Liu and Chen (2006)	69.2 ± 7.3	69.1 ± 7.9	31	40	31.82 ± 12.73	32.19 ± 6.94	China
Lovati et al. (2007)	76.6 ± 7.5	67.6 ± 7.2	108	76	8.20 ± 5.32	15.56 ± 7.93	Italy
Koseoglu and Karaman (2007)	78.3 ± 4.1	76.1 ± 3.9	51	40	21.40 ± 4.39	28.09 ± 3.40	Turkey
Hagnelius et al. (2008)	72.7 ± 10.1	64.1 ± 9.5	42	73	11.2 ± 4.9	13.4 ± 5.8	Sweden
Galimberti et al. (2008)	78.45 ± 4.63	70.13 ± 3.01	29	23	8.63 ± 2.81	19.82 ± 6.16	Italy
Karimi et al. (2009)	75 ± 16	68 ± 8	51	49	14.50 ± 6.57	15.85 ± 8.61	Iran
Villa et al. (2009)	70.8 ± 7.8	74.7 ± 6.7	20	18	16.8 ± 4.7	19.0 ± 4.1	Italy
Linnebank et al. (2010)	73 ± 8	62 ± 10	60	60	15.62 ± 7.04	14.05 ± 7.74	Germany
Agarwal et al. (2010)	65.03 ± 2.1	48.65 ± 1.2	32	127	14.98 ± 2.61	15.7 ± 2.67	India
Morillas-Ruiz et al. (2010)	76.5 ± 3.5	79 ± 4	52	48	21.8 ± 8.7	28.8 ± 7.7	Spain
Faux et al. (2011)	78.4 ± 8.7	70 ± 7	205	760	29.35 ± 1.01	30.29 ± 0.46	Australia
Arlt et al. (2012)	73.5 ± 7.4	50.0 ± 16.8	51	98	16.99 ± 8.83	13.59 ± 7.36	Germany
Almeida et al. (2011)	70 ± 5.93	67 ± 4.45	40	49	16.00 ± 13.01	21.46 ± 13.93	Brazil
Czapski et al. (2012)	—	—	204	99	19.82 ± 17.89	19.43 ± 8.51	Poland
Kim et al. (2013)	79.4 ± 6.8	71.4 ± 6.6	100	121	12.91 ± 10.6	13.81 ± 9.2	South Korea
Kim and Lee (2014)	76.73 ± 7.63	75.86 ± 5.74	77	37	23.24 ± 15.40	30.37 ± 17.12	South Korea
Mansoori et al. (2014)	66.3 ± 8.9	63.8 ± 8.2	80	120	16.53 ± 7.93	19.93 ± 8.15	India
Li et al. (2014)	76.96 ± 7.58	75.14 ± 12.98	126	120	10.86 ± 3.96	15.92 ± 4.45	China
Chen et al. (2015)	67.6 ± 7.9	66.7 ± 6.2	115	115	12.23 ± 8.15	17.21 ± 7.47	China
Cascalheira et al. (2015)	75.1 ± 3.1	71.0 ± 3.0	27	28	23.3 ± 1.88	25.8 ± 1.93	Portugal
Zhong et al. (2016)^*^	69.65 ± 8.63	71.24 ± 6.58	27	23	7.16 ± 1.43	14.81 ± 2.51	China
Zhong et al. (2016)^**^	70.55 ± 8.36	70.28 ± 7.25	31	31	10.10 ± 5.14	18.05 ± 5.55	China
Moretti et al. (2017)	77.9 ± 2.01	76.4 ± 2.3	86	567	5.44 ± 0.68	14.50 ± 0.45	Italy
Ma et al. (2017)	74.62 ± 8.01	72.82 ± 8.87	89	115	11.62 ± 8.09	15.92 ± 8.34	China

* *Altitude 3,380 m*;

** *Altitude 2,260 m*.

The funnel plots appeared to be asymmetrical (Figure 2B). Moreover, Egger's test was performed. The *P*-value was 0.004, indicating there was potential publication bias across all included studies.

### Folate Defiency/Possible Deficiency Is Associated With the Risk for AD: Results From Case-Control Studies

Fifteen case-control studies involving 971 AD patients and 1,059 controls were included in the meta-analysis (Table 2). The sample size ranged from 27 to 181. Eight studies were conducted in European countries and seven studies were conducted in Asian countries.

**Table 2:** Summary of studies regarding the association between folate level and the risk of AD in 14 case-control studies.

**Folate level (nmol/L)**	**References**	**Sample size**		**Mean age (years)**		**OR (95% CI)**	**Adjusted**	**NOS**	**Country**
		**AD**	**Control**	**AD**	**Control**				
13.5 inclusive	Clarke et al., 1998	164	108	73.2 ± 8.6	72.8 ± 8.8	2.3 (1.40, 4.50)^*^	YES	6	UK
	Hogervorst and Smith, 2002	66	62	77 ± 8	76 ± 8	0.41 (0.18, 0.90)	NO	6	UK
	Anello et al., 2004	180	181	71.0 ± 6.6	69.5 ± 12.7	0.95 (0.91, 1.0)	NO	6	Italy
	Mansoori et al., 2014	80	120	66.3 ± 8.9	63.8 ± 8.2	2.4 (1.4, 4.5)	NO	6	India
	Cascalheira et al., 2015	28	27	75.1 ± 1.5	71.0 ± 1.5	0.98 (0.93, 1.04)	NO	8	Portugal
	Ma et al., 2017	89	115	74.62 ± 8.01	72.82 ± 8.87	2.04 (0.53, 6.71)^$^	YES	8	China
≥13.5	Clarke et al., 1998	—	—	—	—	0.7 (0.4, 1.5)^**^	YES	6	UK
	Clarke et al., 1998	—	—	—	—	1.0 (0.5, 1.7)^***^	YES	6	UK
	Quadri et al., 2004	74	55	79.1 ± 7.7	75.6 ± 8.5	2.1 (0.6, 6.8)^#^	YES	6	Switzerland
<13.5	Quadri et al., 2004	—	—	—	—	3.5 (1.1, 11.2)^##^	YES	6	Switzerland
	Mizrahi et al., 2004	75	155	88 ± 7.0	76 ± 7.0	1.3 (0.5, 3.7)^¤^	NO	6	Israel
	Mizrahi et al., 2004	—	—	—	—	1.6 (0.6, 4.2)^¤¤^	NO	6	Israel
	Kim et al., 2013	100	121	79.4 ± 6.8	71.4 ± 6.6	2.70 (1.22, 5.98)	YES	6	South Korea
	Chen et al., 2015	115	115	67.6 ± 7.9	66.7 ± 6.2	2.2 (0.9-5.5)	NO	8	China
	Ma et al., 2017	—	—	—	—	3.42 (1.15, 8.34)^$$^	YES	8	China

* *Plasma folate ≤ 17.1 nmol/L*;

** *plasma folate 17.2–24.2 nmol/L*;

*** *plasma folate >24.2 nmol/L*.

# *Plasma folate 13.5–19.5 nmol/L*;

## *plasma folate <13.5 nmol/L*.

¤ *Plasma folate 7.87–11.40 nmol/*;

¤¤ *plasma folate ≤ 7.86 nmol/L*.

$ *Plasma folate 6.80–15.90 nmol/L*;

$$ *plasma folate ≤ 6.80 nmol/L*.

**Figure 3:**
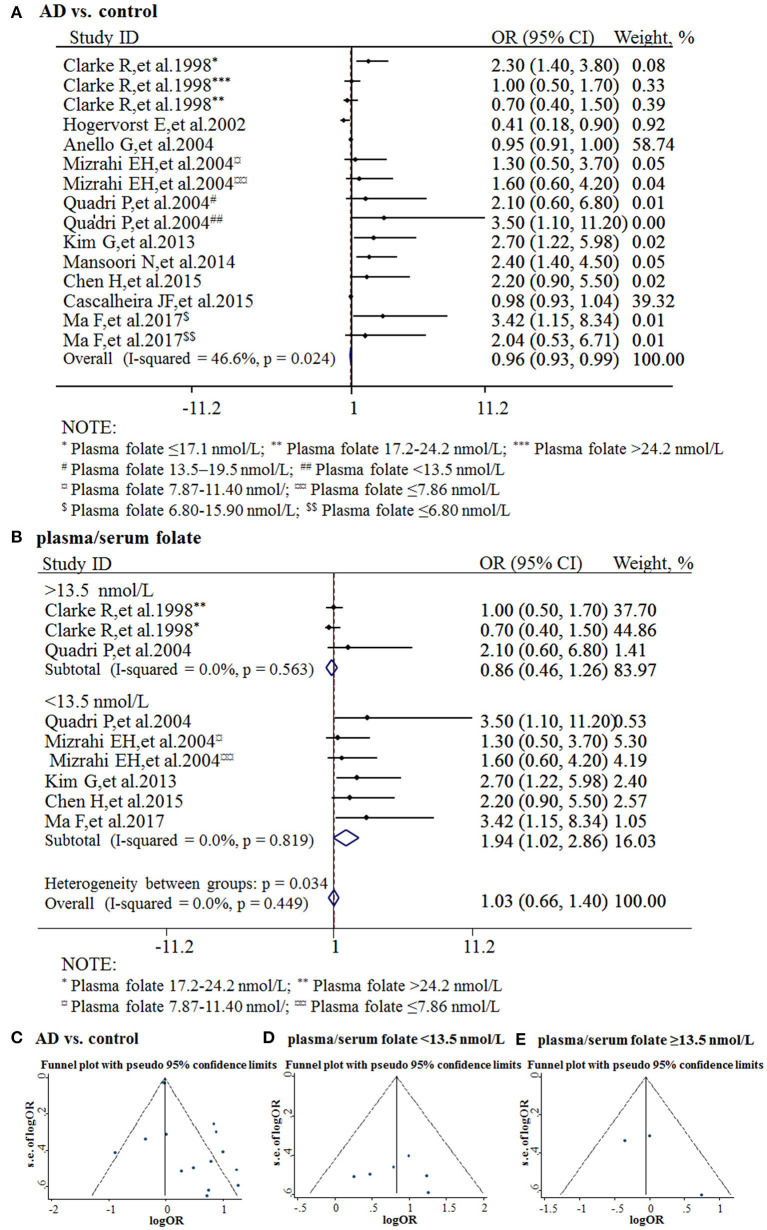
Meta-analysis for the associations between folate levels and risk of AD. **(A)** Combined folate OR of AD patients; **(B)** Combined OR in the folate deficiency/possible deficiency group and normal group; **(C)** Funnel plot for publication bias of AD vs. control; **(D)** Funnel plot for publication bias of the folate deficiency/possible deficiency group; **(E)** Funnel plot for publication bias of the normal folate group.

The combined OR was 0.96 with 95% CI (0.93, 0.99) (Figure 3A). According to the level of plasma/serum folate, all individuals were further divided into two subgroups, folate deficiency/possible deficiency group and normal group with folate level <13.5 and ≥13.5 nmol/L, respectively (WHO, 2015). In the folate deficiency/possible deficiency group, the combined OR was 1.94 (95% CI: 1.02, 2.86) (Figure 3B). However, the combined OR was 0.86 (95% CI: 0.46, 1.26) in the normal group (Figure 3B). The above data indicated that folate deficiency/possible deficiency is correlated with AD risk. It suggested that folate deficiency/possible deficiency may increase the risk for AD.

The funnel plots appeared to be symmetrical and all studies were within the 95% CIs, visually indicating there was no publication bias (Figures 3C–E). An Egger's test was further performed to evaluate publication bias. The *P*-value was 0.011 in all studies, indicating there was publication bias, language bias, inflated estimates, and/or a lack of publications with opposite results. Moreover, the *P*-value was 0.957 in the subgroup of folate deficiency/possible deficiency, indicating there was no publication bias in this subgroup.

### Folate Defiency/Possible Deficiency Increases the Risk for AD: Results From Cohort Studies

**Figure 4:**
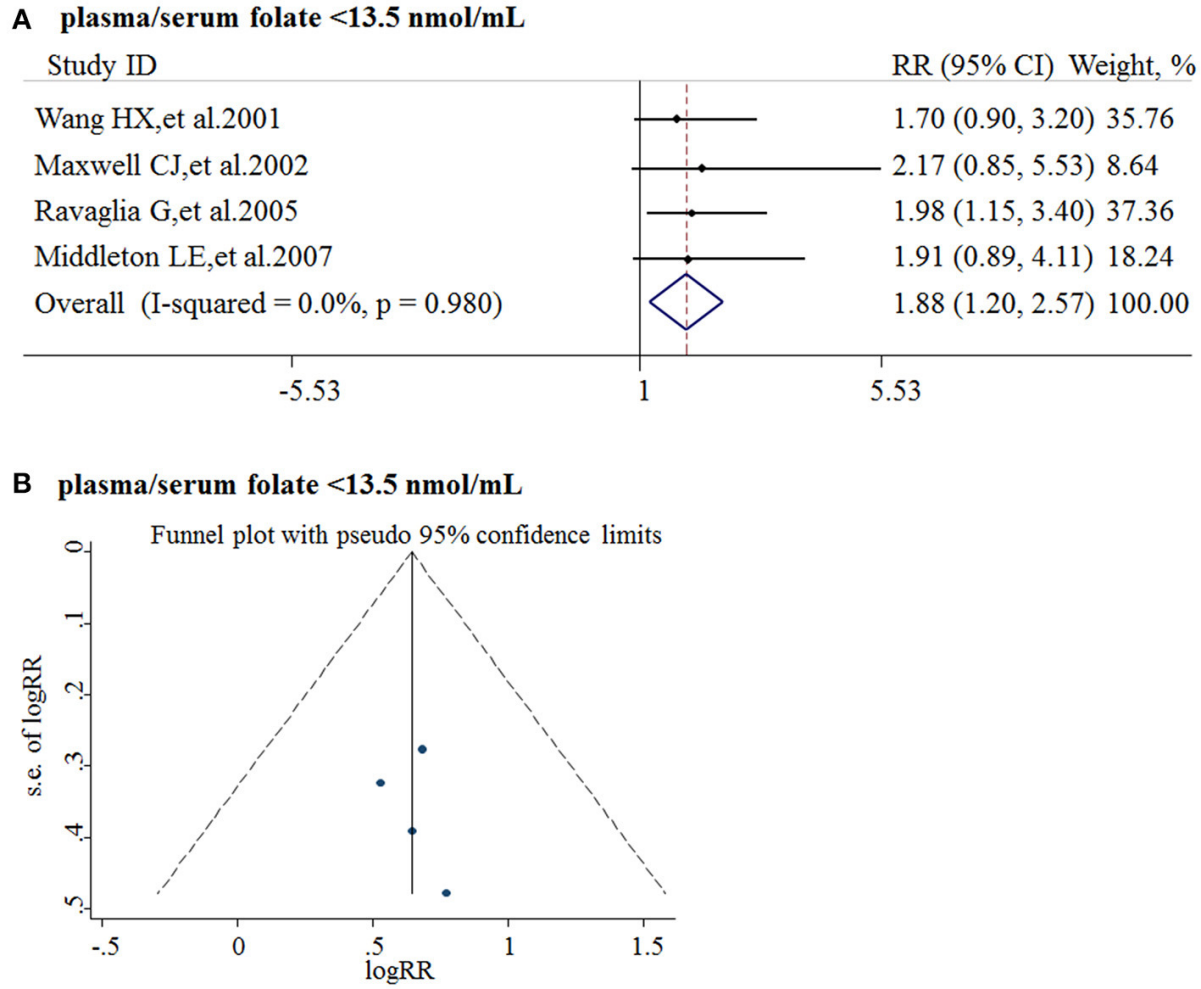
Meta-analysis for the associations between folate levels and risk of AD in general population. **(A)** Combined RR of AD in the folate deficiency/possible deficiency group; **(B)** Funnel plot for publication bias.

Five studies from 2001 to 2017 were included in the meta-analysis (Table 3). Sample size ranged from 190 to 816. Two studies were conducted in Canada, and three in Italy, Sweden, and Switzerland, respectively. All the cohort studies presenting the folate levels of participants were divided into the folate deficiency/possible deficiency group and the normal group with folate level <13.5 and ≥13.5 nmol/L, respectively. In the folate deficiency/possible deficiency group, the combined RR was 1.88 (95% CI: 1.20, 2.57) (Figure 4A). The data indicated that folate deficiency/possible deficiency increases the risk for AD.

**Table 3:** Summary of studies regarding the association between folate level and the risk of AD in five cohort studies.

**Serum folate** ** (nmol/L)**	**References**	**Sample size**	**Follow-up time** ** (years)**	**RR (95% CI)**	**Adjusted**	**NOS**	**Country**
<13.5	Wang et al., 2001	370	3	1.7 (0.9, 3.2)	YES	6	Sweden
	Maxwell et al., 2002	226	5	2.17 (0.85, 5.53)	NO	6	Canada
	Ravaglia et al., 2005	816	4	1.98 (1.15, 3.40)	YES	6	Italy
	Middleton et al., 2007	233	5	1.91 (0.89, 4.11)	YES	6	Canada
≥13.5	Quadri et al., 2005	190	—	1.8 (0.7, 4.5)	YES	6	Switzerland

Publication bias was analyzed by funnel plots. The funnel plots appeared to be symmetrical and all studies were within the 95% CIs, visually indicating there was no publication bias (Figure 4B).

### Sufficient Intake of Folate Reduces the Risk for AD: Results From Cohort Studies

Eleven cohort studies published from 2005 to 2009 were included in the meta-analysis (Table 4). Sample size ranged from 192 to 727. All studies were conducted in the USA.

**Table 4:** Summary of studies regarding the association between folate intake and the risk of AD in 11 cohort studies.

**Folate intake (μg/d)**	**References**	**Sample size**	**Statistical index**	**Follow-up time(years)**	**RR/HR (95% CI)**	**Adjusted**	**NOS**	**Country**
<400	Morris et al., 2006	205	OR	3	1.0 (0.4, 2.3)^*^	YES	6	USA
	Morris et al., 2006	221	OR	3	1.9 (0.7, 5.0)^**^	YES	6	USA
	Luchsinger et al., 2007	192	HR	1.5	0.9 (0.6, 1.3)^#^	YES	6	USA
≥400	Corrada et al., 2005	203	RR	9.3	0.41 (0.22, 0.76)	YES	6	USA
	Morris et al., 2006	195	OR	3	2.7 (1.0, 7.1)^***^	YES	6	USA
	Morris et al., 2006	210	OR	3	1.6 (0.5, 5.2)^****^	YES	6	USA
	Luchsinger et al., 2007	192	HR	1.5	0.5 (0.3, 0.9)^##^	YES	6	USA
	Nelson et al., 2009	727	HR	9	1.14 (0.71, 1.84)^§^	YES	6	USA
	Nelson et al., 2009	727	HR	9	0.95 (0.54, 1.66)^§§^	YES	6	USA
	Nelson et al., 2009	727	HR	9	1.36 (0.68, 2.72)^§§§^	YES	6	USA
	Nelson et al., 2009	726	HR	9	1.74 (0.80, 3.83)^§§§§^	YES	6	USA

* *Total folate intake 240–304 μg/d*;

** *total folate intake 304–392 μg/d*;

*** *total folate intake 392–620 μg/d*;

**** *total folate intake 621–1,660 μg/d*.

# *Total folate intake ≤ 292.9 μg/d*;

## *total folate intake ≥487.9 μg/d*.

§ *Total folate intake 430 μg/d*;

§§ *total folate intake 476 μg/d*;

§§§ *total folate intake 524 μg/d*;

§§§§ *total folate 698 μg/d*.

**Figure 5:**
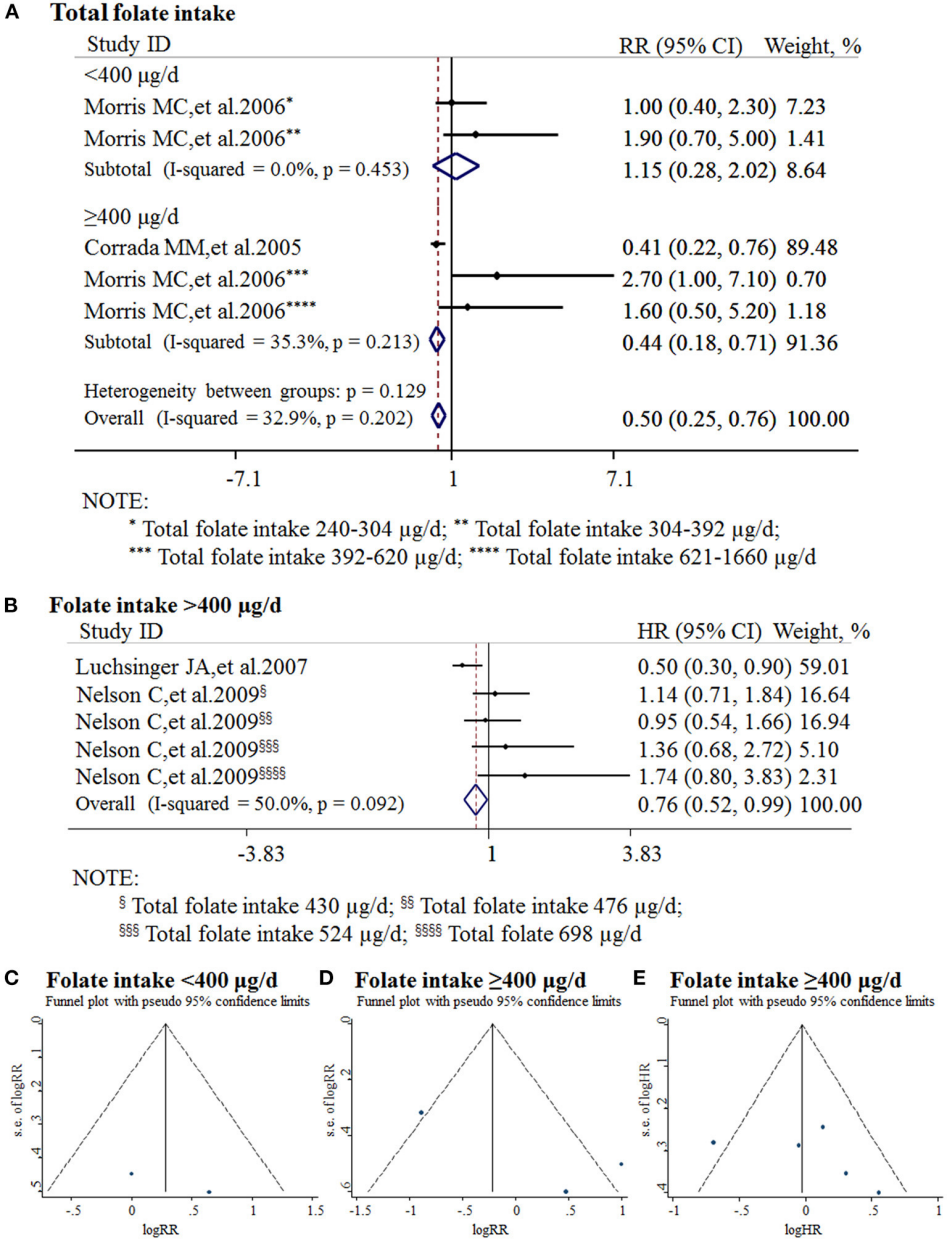
Meta-analysis for the associations between daily intake of folate and the risk of AD in the general population. **(A)** Combined RR of AD with insufficient and sufficient folate intake, respectively; **(B)** Combined HR of AD with sufficient folate intake; **(C)** Funnel plot for publication bias of insufficient folate intake; **(D)** Funnel plot for publication bias of sufficient folate intake (combined RR); **(E)** Funnel plot for publication bias of sufficient folate intake (combined HR).

All the 11 cohort studies were included in the meta-analysis to examine the association between folate intake and AD in the elderly. The combined RR was 0.50 (95% CI: 0.25, 0.76). As the recommended daily allowance of folate is 400 μg, a daily intake of 400 μg of folate was used as the cut-off to define the two subgroups (Benoist, 2008). When the daily intake of folate was <400 μg, the combined RR and HR were 1.15 (95% CI: 0.28, 2.02) and 0.9 (95% CI: 0.6, 1.3), respectively (Figure 5A). When the daily intake of folate was equal to or higher than 400 μg, the combined RR and HR were 0.44 (95% CI: 0.18, 0.71) and 0.76 (95% CI: 0.52, 0.99), respectively (Figures 5A,B). It indicated that sufficient folate intake (i.e., ≥400 μg/d) is a protective factor for AD, which significantly reduces the risk for AD.

Publication bias was analyzed by funnel plots. The funnel plots appeared to be symmetrical and all studies were within the 95% CIs, visually indicating there was no publication bias (Figures 5C–E).

## Discussion

The present analyses were designed to comprehensively evaluate the associations between AD and folate levels. The pooled results showed that the folate level of AD patients was lower compared with that of healthy controls. Moreover, the deficiency/possible deficiency of folate (<13.5 nmol/L) increases the risk for AD. Importantly, sufficient daily intake of folate (≥400 μg/d) reduces the risk of AD occurence.

A meta-analysis was performed to explore the association between folate and Alzheimer's disease based on studies published before January, 2014 (Shen and Ji, 2015). Compared with the previous one, our study has the following distinguished characteristics. First, inclusion criteria of this study was restricted to AD, but not all forms of dementia. It might minimize the heterogeneity of different studies and make the conclusion more convincing. Moreover, cohort studies were included in addition to case-control studies. It indicated that folate deficiency/possible deficiency may have a causal effect on AD development. In addition, subgroup analysis was performed to evaluate the association between folate and AD. It definitely minimized the heterogeneity between the two populations, i.e., folate within normal range and folate deficiency/possible deficiency. Thus, our study highly indicated that folate deficiency/possible deficiency is a risk factor for AD. Importantly, the combined effect sizes of daily intake of folate and AD were first analyzed in this study. It indicated that sufficient daily intake of folate significantly reduces the risk for AD. Furthermore, the included studies were updated and cross-sectional studies were also included. For example, 52 cross-sectional studies covering 3,496 AD patients and 4,318 controls were included in the current study. The increase of the sample size makes the difference between AD and controls more reliable.

There were some limitations in this study. First, the different classification criteria, different categories of demographic characteristics, and different follow-up periods of the included studies may affect the pooled effects. Moreover, most of the studies were from North America, Europe, and Asia, with limited data from Africa and South America. The regional effect might need to be considered. Furthermore, more long-term follow-up studies were needed to confirm the preventive effect of sufficient folate intake on AD.

Although the mechanism of folate protecting against AD is not clear, there are plausible explanations for the association between AD and folate levels. First, folate has important biological activities, such as anti-oxidative stress, which counteracts AD development (Alonso et al., 2001; Agnati et al., 2007; De Felice et al., 2007; Zhang et al., 2009). Secondly, folate participates in the DNA methylation process as a donor of methyl, while DNA methylation plays a crucial role in aging and AD pathogenesis (Zhang et al., 2009; Smith and Lunnon, 2017). In addition, folate regulates the expression of both β-secretase and γ-secretase, theses two key secretases contribute to Aβ generation and neruitic plaque formation (Fuso et al., 2007; Ly et al., 2013; Wang et al., 2017; Zhang et al., 2020). Moreover, folate inhibits tau phosphorylation and subsequent neurofibrillary tangle formation by indirectly regulating the activity of protein phosphatase cyclin-dependent kinase and glycogen synthase kinase (Sontag and Sontag, 2014). Furthermore, folate might also be implicated in AD by regulating the level of homocysteine (Hcy) as it is a co-factor of Hcy metabolism (Zhang et al., 2009; Mccaddon and Miller, 2015; Smith and Refsum, 2016; Smith et al., 2018).

## Conclusions

AD patients had lower levels of folate than healthy controls. Folate deficiency/possible deficiency may increase the risk for AD. Sufficient daily intake of folate may reduce the risk of AD occurrence. Trials have already shown that folic acid supplementation can slow cognitive decline and brain atrophy in patients with mild cognitive impairment (De Jager et al., 2012; Douaud et al., 2013; Ma et al., 2019). These findings indicated that sufficient folate intake is preventive against AD. Randomized controlled trials are needed to verify the causality of sufficient folate intake or folic acid supplementation and AD prevention.

## Author Contributions

YW designed the study. XZ, GB, and YY searched for and screened the literature. XZ, GB, and YY extracted the data independently. XZ, DL, GB, and YL conducted the meta-analysis constructions. XZ, GB, and YY analyzed the data and wrote this manuscript. XL, YL, and YW revised the manuscript. All authors reviewed and proved the manuscript.

## Conflict of Interest

The authors declare that the research was conducted in the absence of any commercial or financial relationships that could be construed as a potential conflict of interest.
